# An Approach for the Customized High-Dimensional Segmentation of Remote Sensing Hyperspectral Images

**DOI:** 10.3390/s19132887

**Published:** 2019-06-29

**Authors:** Blanca Priego, Richard J. Duro

**Affiliations:** 1 Biomedical Engineering and Telemedicine Researching Group, University of Cádiz, 11002 Cádiz, Spain; 2 Institute of Research and Innovation in Biomedical Sciences of the Province of Cádiz (INiBICA), University of Cádiz, 11002 Cádiz, Spain; 3 Integrated Group for Engineering Research, Centro de Investigación en Tecnologías de la Información y las Comunicaciones (CITIC), Universidade da Coruña, 15403 Ferrol, Spain

**Keywords:** hyperspectral image classification, cellular automata, evolutionary algorithm, hyperspectral image segmentation, differential evolution, remote sensing

## Abstract

This paper addresses three problems in the field of hyperspectral image segmentation: the fact that the way an image must be segmented is related to what the user requires and the application; the lack and cost of appropriately labeled reference images; and, finally, the information loss problem that arises in many algorithms when high dimensional images are projected onto lower dimensional spaces before starting the segmentation process. To address these issues, the Multi-Gradient based Cellular Automaton (MGCA) structure is proposed to segment multidimensional images without projecting them to lower dimensional spaces. The MGCA structure is coupled with an evolutionary algorithm (ECAS-II) in order to produce the transition rule sets required by MGCA segmenters. These sets are customized to specific segmentation needs as a function of a set of low dimensional training images in which the user expresses his segmentation requirements. Constructing high dimensional image segmenters from low dimensional training sets alleviates the problem of lack of labeled training images. These can be generated online based on a parametrization of the desired segmentation extracted from a set of examples. The strategy has been tested in experiments carried out using synthetic and real hyperspectral images, and it has been compared to state-of-the-art segmentation approaches over benchmark images in the area of remote sensing hyperspectral imaging.

## 1. Introduction

Even though quite a few methods have been proposed in the last two decades for hyperspectral image classification, addressing this task continues to be a challenging problem. There exist some particular aspects of the segmentation and classification of hyperspectral images that complicate the partitioning or labeling of the data. The first concern emerges when answering the question of *what is considered a homogeneous region or a class?* Images may be segmented or classified in different ways as a function of what the user wants to see or highlight. That is, a hyperspectral image may be segmented into regions corresponding to fields, urban areas and water or into more detailed regions such as different types of vegetation, distinguish roads from buildings or even separate different types of water bodies. This introduces a level of subjectivity linked to the interpretation of the data that needs to be contemplated in the process of implementing automatic classifiers. As a consequence, classification algorithms should display the capability of adapting to meet the classification needs of different users or final applications, but this is not usually the case.

Furthermore, methodological problems arise when processing hyperspectral data. The high dimensionality of the images and the very limited availability of reliably labeled images complicate the spatial partitioning. Increasing the number of spectral bands in hyperspectral images requires that the number of labeled training pixels grow exponentially in order to preserve statistical integrity for supervised classification purposes [1]. Additionally, hyperspectral images are highly correlated in the spatial and spectral dimensions. It is essential to construct spectral-spatial segmentation/classification techniques that can consider within their processing dependencies that may exist among neighboring pixels. This complicates the design of new algorithms and compromises their efficiency.

Finally, practical and operational problems also arise when processing hyperspectral data. First, and related to the first methodological problem, acquiring hyperspectral data and building reliable labeled datasets are costly in terms of economics, time and resources. There are very few publicly available labeled hyperspectral datasets. When they exist, they are often of dubious reliability with respect to their labels. Therefore, it is difficult to produce training sets reflecting the desires of the user in terms of classification. Moreover, the acquisition process is likely to be corrupted by distortions and noise. Atmospheric and illumination variations may spoil the dataset, making it necessary to design robust methods to segment and classify hyperspectral images. Finally, most current algorithms typically require expert knowledge in order to provide an initial parameter set-up to adapt them to a particular problem or application, limiting their practical usability.

To address these issues, in this work, we propose a complete methodology for conducting a multi-stage classification procedure based on a segmentation step followed by a subsequent classification stage. Here, we concentrate on the segmentation step and use off the shelf classifiers (SVMs) just as a means to produce final classification results that can be compared to other approaches in the literature. The objective is to make the segmentation more adapted to user needs and simpler to obtain by providing a general methodology that starts with a process for easily constructing low dimensional training sets that reflect the type of segmentation the user desires over a given type of high dimensional images. These training data sets are used within an evolutionary segmenter generator, called ECAS-II to produce Cellular Automata (CA) based segmenters that conform to a type of structure we have called Multi-gradient based Cellular Automata (MGCA). MCGAs are able to segment multidimensional images without any projection onto lower dimensions. The conjunction of all of these steps leads to a workflow that allows obtaining hyperspectral image segmenters using very small training sets. These segmenters conform to the user needs in terms of how she wants the image to be segmented, making use of all of the information available in the images.

The remainder of the paper is structured as follows. The next section provides a brief review of related work on hyperspectral image segmentation. The MGCA Cellular Automata structure considered in this paper is presented in Section 3. Section 4 is devoted to the detailed description of the operation of the ECAS-II algorithm. Then, in Section 5, we introduce how the training sets are constructed and, in Section 6, the classification algorithm that will be used for the purpose of comparing the results obtained to those found in the literature is described. Experimental results of the proposed classification algorithm are presented in Section 7, including a performance comparison to other existing methods. Finally, some concluding remarks and perspectives are discussed in Section 8.

## 2. Related Work

The earliest hyperspectral image classification methods address the problem from a pixel-wise perspective. This means that each pixel is processed independently without considering contextual (spatial) information. Among these traditional classification methods, which have been used since the 1990s, we may find maximum-likelihood (ML) based methods [2], multinomial logistic regression based techniques (MLR) [3], Fisher’s linear discriminant analysis (LDA) [4], Linearly Constrained Discriminant Analysis (LCDA) [5], shape filtering [6], Artificial Neural Networks (ANN) [7] and fuzzy methods [8]. In [9], a Support Vector Machine (SVM) technique was applied to hyperspectral data for the first time. The SVM algorithm seeks the optimal separation surface between classes. It does this by identifying the most representative training sample of each class, called support vector. SVM has shown very good performances for classifying high-dimensional data in situations with small training sets. Furthermore, a feature selection step to reduce the dimensionality of the image cube is not required [10,11,12,13]. In [14,15], a K-nearest neighbor method is applied. These well-known types of methods seek a predefined number of training samples that are close to the test sample and assign a label to the test sample that reflects the majority category label of these k-nearest training samples.

The aforementioned methods consider hyperspectral data as a set of spectral measurements without meaningful arrangement. This means that each spectral vector will be classified in the same manner regardless of its spatial position within the hyperspectral cube. This is the reason why pixel-wise classifiers often produce classification maps with “salt and pepper” noise. However, taking into account correlations between spatially or temporally neighboring pixels may significantly improve the performance of the classification algorithm. Methods that follow this methodological line are called spectral-contextual techniques [16].

The design of spectral-contextual methods, hereafter spectral-spatial methods when considering spatial correlations, as is the case in this paper, normally entails the application of a multi-stage classification procedure. There are mainly two ways to include spatial information. The first one processes each pixel using spatial information within a window of fixed/adaptive size centered on the pixel under analysis. The second approach first segments the image into homogeneous regions, and then assigns a classification label to each of the regions.

One of the earliest methods to follow the first approach employed Markov Random Fields (MRF) in conjunction with an adaptive ML [17]. Markov random fields imply that the conditional distribution of a point in the field only depends on its neighbors. Different order neighborhoods can be defined, from the first-order neighborhood of a pixel, usually defined as the four pixels surrounding it, to any other order by adding corner pixels to a given order neighborhood system. In [17], the authors show that this method improves the performance of an ordinary ML technique. In addition, in [18], a Markov random field regularization is applied to the result of a pixel-wise classification of hyperspectral images using a probabilistic support vector machine. In addition, in [19], an iterative procedure is applied with the objective of introducing spatial relaxation while preserving an accurate representation of the edges of class boundaries of a first-local neighbors approach, both as a preprocessing step and to post process the result of an MLR model. However, using a standard neighborhood may not be sufficient to contain enough spatial information on the structures present in the image and increasing the size and shape of the spatial window is, in most cases, computationally intractable.

The extraction of spatial and spectral features has also been addressed by the application of N-dimensional filters to principal-component subspaces. They have been proven efficient when two-dimensional [20] and three-dimensional [21] Gabor filters are considered, or a three-dimensional discrete wavelet transform (3DDWT) [22,23] is applied to the hyperspectral data, providing robustness against additive white Gaussian noise.

Additionally, a number of techniques have used a local binary pattern (LBP) [24] to encapsulate the spatial structure of local image texture information for rotation invariant texture classification. In a further step, the authors of [25] couple an LBP-driven class-conditional probability with an MRF-driven prior probability in a maximum a posteriori (MAP) Bayesian formulation, which leads to high classification accuracy while being robust under a small training sets and Gaussian-noise conditions.

Several proposals based on mathematical morphology have been widely addressed in the literature. These techniques are focused on image structural information, which is obtained through the application of morphological operators (erosion/dilation, opening/closing, top-hat/bot-hat, rank filters, etc.). Morphological operators are applied to each pixel using a multi-scale approach, thus obtaining what is called Morphological Profiles (MP), which serve as inputs to a posterior classification step. The first works based on MP were applied over panchromatic images [26,27]. Afterwards, in [28,29,30], the approach was extended to hyperspectral images by building Extended Morphological Profiles (EMP) from several first components of a Principal Component Analysis (PCA) [31] applied to the data cube. Later, in [32], the same procedure of reducing the dimensionality of hyperspectral images through a PCA or a Minimum Noise Fraction (MNF) transform is followed. In [33,34,35,36], the concept of MP and EMP was enhanced by extracting additional spatial features, leading to the notion of Attribute Profiles (AP).

Another related technique makes use of SVM with composite kernels (SVM-CKs). A pixel-wise classification is performed in which spatial information is introduced through an operation involving spatial and spectral kernels as well as combined spatial-spectral kernels. In SVM-CKs, the spatial feature is usually expressed as the mean or standard deviation of the gray value distribution of pixels in a fixed spatial neighborhood [37,38]. A framework for generalized-composite-kernel-based classification is proposed in [39]. It differs from the previous ones in that it relies on a Sparse Multinomial Logistic Regression (SMLR) classifier to produce the final classification results and in modeling the spatial information using Extended Multi-attribute Profiles (EMAPs).

Generally, mathematical morphology based approaches provide good classification accuracy results, but most of the methods still struggle with the high dimensionality of the data. Morphological operators were initially created for single-band images, and, generally, their extension to hyperspectral multidimensional data has been addressed by independently extracting and processing one or a few representative grayscale images, leading to significant information loss. Furthermore, the features extracted with these operators usually imply a great deal of tuning to adapt them to a particular data set.

An alternative to the extraction of handcrafted features is to train a data structure so that it can then obtain relevant features straight from the pixels [40]. Training deep convolutional neural networks (CNNs) [41,42] is a popular example of this. The main drawback of CNNs is that they require very large labeled data sets for training, which is a problem taking into account the lack of labeled hyperspectral data. To tackle this issue, authors in [43] use unsupervised learning to train two models based on multiscale independent component analysis (MICA) and a stacked convolutional autoencoder (SCAE). They extract features from large sets of unlabeled data, and, as is mostly the case for these types of approaches, they assume that the features produced by the learned models can be generalized.

The second procedure for the inclusion of spatial information in spectral-spatial classification methods starts with a preliminary spatial/spectral clustering/segmentation stage over which an area-fusion stage is performed based on supervised criteria. Segmentation is defined as an exhaustive partitioning of an image into non-overlapping regions. Each of these regions is taken to be homogeneous in terms of some criterion of interest. Thus, a segmentation step provides regions of connected pixels that share particular properties. Afterwards, the classification is carried out for the regions using any of the multiple classification methods, such as SVM. The key to this approach lies in the segmentation step, which is basically where the spectral-spatial integration is performed. This step has been addressed through different techniques: ECHO [44], partitional clustering methods [45], watershed transformation [46], hierarchical segmentation (HSeg) or region growing [47]. The classification accuracy of the previously cited works is conditioned by the performance of the selected segmentation approach, which is normally highly sensitive to the measure of region homogeneity and the inherent parameters of the segmentation algorithm [48].

The above-mentioned spectral-spatial classification methods have been shown to improve on the performance of pixel-wise techniques and provide more homogeneous classification maps. However, some of the approaches are based on fixed rules, or, at best, need extensive hand tuning of parameters in order to adapt the segmentation detail to the user needs. On the other hand, those algorithms that learn useful features directly from the dataset require large labeled datasets or project the high-dimensional image onto a lower dimension, leading to the possible loss of valuable information.

In this work, we propose a complete methodology for conducting a multi-stage classification procedure that adheres to this last approach that is, it uses a segmentation step followed by a subsequent classification stage. The main objective of this methodology is to provide a means to make the segmentation step more adapted to user needs and simpler to obtain as well as to improve it. To this end, we will introduce the ECAS-II evolutionary segmenter generator as well as a Cellular Automata (CA) based segmenter structure that we have called Multi-gradient based Cellular Automata (MGCA), which is able to segment multidimensional images without any projection onto lower dimensions. ECAS-II provides the transition rule set for the Cellular Automata based segmenters adapted to particular segmentation needs as a function of a set of low dimensional training images in which the user expresses the segmentation requirements of the application. This approach differs from the algorithms presented above in that it does not provide as an output a labeled segmentation map or classification. Instead, it performs an image “regularization” or segmentation that is adapted to the particular segmentation level required by the user. Thus, its function is to adapt the hyperspectral image to a particular classification purpose. Once the original hyperspectral image is transformed by the ECAS-II generated CA and thus fitted to a particular type of segmentation, any pixel-wise classification algorithm can be applied to label the regions of the image and, consequently, finalize the classification process. For the examples in this particular paper, we have selected for this classification stage a multi-class pairwise (one versus one) SVM classification algorithm, which is directly applied to the segmented data cube. Nevertheless, for this classification step, other suitable algorithms could be considered, such as, for instance, Extreme Learning Machines (ELM) [49,50].

Using ECAS-II and the CAs it generates for segmentation improves on previous approaches in that it is capable of simultaneously fulfilling the following aspects:

- It can adapt to the particular segmentation grain or detail required by the user.
- The training procedure is performed without a need for large training or labeled sets of real images. It makes use of a methodology that simplifies the training process by using low dimensional reference samples. This methodology also reduces expert knowledge requirements.
- It works with the whole spectrum of the images. As it uses a dimensionality independent spectral distance measure, it does not need to project the spectral information onto lower dimensional spaces.
- Its low-level processing operations are intrinsically parallel, making it attractive for concurrent implementation on hardware such as GPUs.
- It is very competitive, as compared to other approaches found in the literature, in terms of performance.

## 3. Multi-Gradient Based Cellular Automata

### 3.1. Cellular Automata

Von Neumann and Ulam [51] proposed CAs as a biologically inspired distributed computing paradigm. A CA is implemented as a regular uni or multidimensional grid of cells, each characterized by a cell state. The CA is iteratively executed so that all cell states are repeatedly updated according to some transition rules. The particular rule to be applied to each cell in a given iteration is selected, taking into account the current state of that cell and the states of the cells in its neighborhood.

The goal of this approach is for the CA to converge to the desired state after its execution for *N* iterations. The key to taking advantage of the great potential of CAs to achieve a particular purpose is to conform its set of transition rules adequately. Unfortunately, the determination of the appropriate rules for the automata is far from straightforward, posing a great challenge to the algorithm designer. It would thus be desirable to be able to automate the process of determining the appropriate transition rules for a particular purpose. This automatic rule selection has been addressed from many different perspectives by the cellular automata community [52], and evolutionary techniques have turned out to be a very popular approach to obtaining the transition rule set [53,54].

Regarding the application of CAs to hyperspectral images, their potential has still hardly been explored. Some authors have designed CA structures to segment low dimensional images [53,54,55], and some work has been carried out in the high-dimensional image processing area in terms of edge detection [56] or image segmentation [57]. However, in these cases, the set of transition rules were hand created ad hoc and, in order to run them over hyperspectral data, the spectral information was usually projected onto a lower dimension using a PCA or MNF transformation.

### 3.2. General Operation of MGCA

Focusing now on the particular CA implementation proposed in this paper and denoted as Multi-Gradient based Cellular Automata (MGCA), let us start by saying that each pixel of the hyperspectral image is handled by one cell of the automaton. The state of the cell ($s_{i}$) is expressed as an *N*-element vector, taking values in the range [0, 1] that correspond to the intensities of different bands of a spectrum. These cells are initialized with the spectra present in their assigned pixel. We will indistinctly talk about cells or pixels when describing the operation of the CA. As a consequence, the state space is continuous and it is represented by the positive $R^{N}$ vector space.

In what follows, we will describe the procedure for gradually and iteratively updating the state of all the cells of the CA so that they converge towards a segmented image. The objective is to homogenize the state vectors, which are basically spectra, corresponding to the cells (pixels) of each one of the different regions into a narrow band of spectra so that any classification algorithm can then easily classify the different regions. It should be noted here that the strategy contemplated for the CA operates over the whole pixel spectrum, thus avoiding the need for dimensionality reductions or projections.

### 3.3. Spectral Distance Measure

Like any cellular automaton, the state of every cell must be updated every iteration. This implies that for each cell one particular rule out of the set of transition rules available to the automaton to control its behavior must be chosen and applied. This choice is made as a function of the state vector (spectrum) of the particular cell and that of its $N_{Smax}\times N_{Smax}$ closest neighboring cells, $N_{Smax}$ being the maximum size of the spatial neighborhood window centered on the cell that will be considered.

The comparison of spectra obviously requires a spectral distance measure. For this algorithm, we have chosen the popular normalized spectral angle (SA) distance measure. The advantage of using the SA is that it makes the measurements independent of the number of spectral components, allowing for the use of the same CA over different images regardless of their spectral dimensionality. Moreover, the SA is also invariant to changes of scale, which makes the method insensitive to changes in illumination. Thus, the normalized spectral angle, $\alpha_{i,j}$ between the spectrum of a cell *i* and a cell *j* is defined as:

(1) $$\alpha_{i,j}=\frac{2}{\pi }cos^{-1}\left(\frac{\sum s_{ij}s_{i}}{\sqrt{\sum s_{ij}^{2}}\sqrt{\sum s_{i}^{2}}}\right).$$

### 3.4. Deciding on the Appropriate Update Rule

As commented on in the previous section, every iteration of a particular transition rule is chosen for application to each cell depending on the spectra of its neighborhood. However, to do this when there are several neighborhood pixels, several spectral angle measures must be somehow combined. Thus, to enhance the descriptive power of the approach, we propose combining neighboring spectral measurements by means of the calculation of spatial gradients, $G_{N_{Si}}$. More concretely, to perform the calculation of the gradients, we chose to take into account the pixels within three different $N_{S}\times N_{S}$ cell windows, with $N_{S}=\left\{3,5,7\right\}$ (in other words, we have used the closest 8, 24 and 48 neighboring cells). Thus, each gradient represents information on spectral change intensity and direction for three resolutions leading to a multi-gradient based decision process.

Two 2D masks, $M_{X_{N_{S}}}$ and $M_{Y_{N_{S}}}$, are considered for obtaining the spatial gradients in each of the $N_{S}\times N_{S}$ windows:

(2) $$G_{N_{S_{i}}}=\left(G_{X_{N_{S_{i}}}},G_{Y_{N_{S_{i}}}}\right),\;N_{S}=\left\{3,5,7\right\},$$

(3) $$\begin{array}{c} G_{X_{N_{S_{i}}}}=\sum_{j=1}^{N_{S}\cdot N_{S}}\alpha_{i,j}\cdot M_{X_{N_{S_{j}}}},\\ G_{Y_{N_{S_{i}}}}=\sum_{j=1}^{N_{S}\cdot N_{S}}\alpha_{i,j}\cdot M_{Y_{N_{S_{j}}}}, \end{array}$$

(4) $$\begin{array}{c} M_{X_{3}}=\frac{1}{2}\cdot \left[\begin{array}{ccc} -\frac{1}{2} & 0 & \frac{1}{2}\\ 1 & 0 & 1\\ -\frac{1}{2} & 0 & \frac{1}{2} \end{array}\right],\;M_{Y_{3}}=M_{X_{3}}^{T},\\ M_{X_{5}}=\frac{10}{33}\cdot \left[\begin{array}{ccccc} -\frac{1}{8} & -\frac{1}{5} & 0 & \frac{1}{5} & \frac{1}{8}\\ -\frac{1}{5} & -\frac{1}{2} & 0 & \frac{1}{2} & \frac{1}{5}\\ -\frac{1}{4} & -1 & 0 & 1 & \frac{1}{4}\\ -\frac{1}{5} & -\frac{1}{2} & 0 & \frac{1}{2} & \frac{1}{5}\\ -\frac{1}{8} & -\frac{1}{5} & 0 & \frac{1}{5} & \frac{1}{8} \end{array}\right],\;M_{Y_{5}}=M_{X_{5}}^{T},\\ M_{X_{7}}=\frac{361}{1527}\cdot \left[\begin{array}{ccccccc} -\frac{1}{18} & -\frac{1}{13} & -\frac{1}{10} & 0 & \frac{1}{10} & \frac{1}{13} & \frac{1}{18}\\ -\frac{1}{13} & -\frac{1}{8} & -\frac{1}{5} & 0 & \frac{1}{5} & \frac{1}{8} & \frac{1}{13}\\ -\frac{1}{10} & -\frac{1}{5} & -\frac{1}{2} & 0 & \frac{1}{2} & \frac{1}{5} & \frac{1}{10}\\ -\frac{1}{9} & -\frac{1}{4} & -1 & 0 & 1 & \frac{1}{4} & \frac{1}{9}\\ -\frac{1}{10} & -\frac{1}{5} & -\frac{1}{2} & 0 & \frac{1}{2} & \frac{1}{5} & \frac{1}{10}\\ -\frac{1}{13} & -\frac{1}{8} & -\frac{1}{5} & 0 & \frac{1}{5} & \frac{1}{8} & \frac{1}{13}\\ -\frac{1}{18} & -\frac{1}{13} & -\frac{1}{10} & 0 & \frac{1}{10} & \frac{1}{13} & \frac{1}{18} \end{array}\right],\\ M_{Y_{7}}=M_{X_{7}}^{T}, \end{array}$$

where $M_{X_{N_{S_{j}}}}$ and $M_{Y_{N_{S_{j}}}}$ correspond to the *j*th components of gradient masks $M_{X_{N_{S}}}$ and $M_{Y_{N_{S}}}$. $G_{N_{S_{i}}}$ is the gradient vector calculated for a window of size $N_{S}\times N_{S}$ located at cell *i*.

A gradient vector can be expressed using its modulus, $|G_{N_{S_{i}}}|$, and an angle value, $\phi_{N_{S_{i}}}$, defined as:

(5) $$\begin{array}{c} {|G}_{N_{S_{i}}}|=\sqrt{G_{X_{N_{S_{i}}}}^{2}+G_{Y_{N_{S_{i}}}}^{2}},\\ \phi_{N_{S_{i}}}=\tan^{-1}\left(\frac{G_{Y_{N_{S_{i}}}}}{G_{X_{N_{S_{i}}}}}\right). \end{array}$$

**Figure 1:**
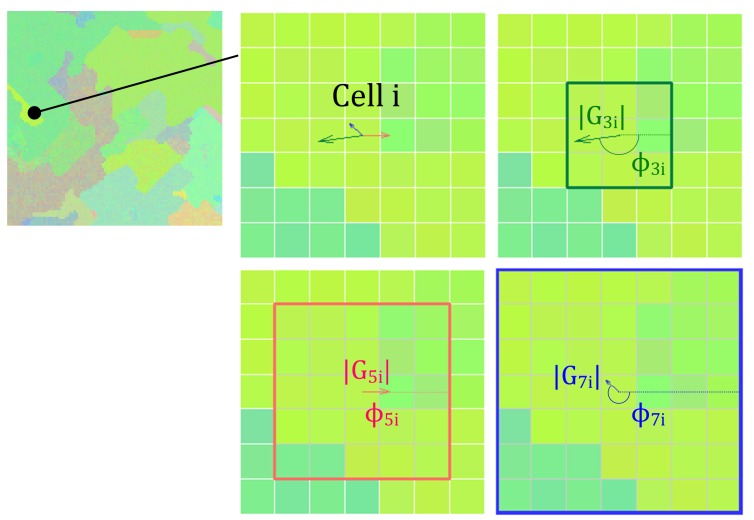
Extraction of the three gradient vectors, $G_{N_{Si}}$, with $N_{S}=\left\{3,5,7\right\}$, for a pixel from a RGB image.

The modulus $|G_{N_{S_{i}}}|$ provides an indication of the amount of change of the spectrum in the direction given by angle, $\phi_{N_{S_{i}}}$, for a window size of $N_{S}$. To illustrate this, Figure 1 depicts an example of extracting the modulus and angle values for a cell from an RGB image. In this CA implementation, the values of the three moduli and angles, ($G_{3_{i}},G_{5_{i}},G_{7_{i}},\phi_{3_{i}},\phi_{5_{i}}\phi_{7_{i}}$), represent the information the CA collects from the neighborhood of a cell *i* in order to decide on the transition rule to apply.

On the other hand, the transition rule set that governs the behavior of the cellular automaton is made up of *M* rules, each one of them represented by a 6-parameter vector. The first five parameters correspond to the condition upon which the decision is made and the last one to the action to be applied:

(6) $$CA=\left\{\begin{array}{cccccc} |G_{r_{3_{1}}}| & |G_{r_{5_{1}}}| & |G_{r_{7_{1}}}| & \phi_{r_{5_{1}}} & \phi_{r_{7_{1}}} & \theta_{r_{{}_{1}}}\\ \vdots & \vdots & \vdots & \vdots & \vdots & \vdots \\ |G_{r_{3_{k}}}| & |G_{r_{5_{k}}}| & |G_{r_{7_{k}}}| & \phi_{r_{5_{k}}} & \phi_{r_{7_{k}}} & \theta_{r_{{}_{k}}}\\ \vdots & \vdots & \vdots & \vdots & \vdots & \vdots \\ |G_{r_{3_{M}}}| & |G_{r_{5_{M}}}| & |G_{r_{7_{M}}}| & \phi_{r_{5_{M}}} & \phi_{r_{7_{M}}} & \theta_{r_{{}_{M}}}. \end{array}\right\}$$

**Figure 2:**
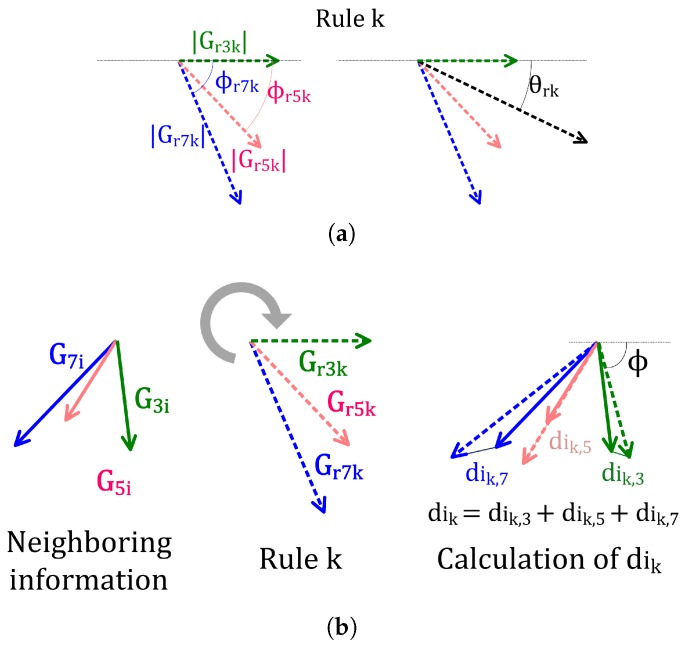
(**a**) Rule representation: (left) condition parameters and (right) action parameter; (**b**) comparison between the neighborhood information of a cell *i* and the condition parameters of a rule *k*.

Notice that the encoding of the transition rules is a direct representation of three vector gradients. To make things clear, here subindex *r* is used to prevent confusions between rule parameters and the values of the gradient vectors that were extracted from the neighborhood information of a cell. Now, if we select a representation where these three vector gradients are independent of angle rotations and reflections, only five parameters are required to represent the three vector set. One of the angles, in this case, $\phi_{r_{3_{k}}}$, will always be taken as equal to 0. Figure 2a (left) shows a geometrical representation of the condition parameters of a rule.

For each iteration, only one out of the *M* possible rules is applied to each cell. The decision of which one is based on comparing the neighborhood information vector ($G_{3_{i}},G_{5_{i}},G_{7_{i}},\phi_{3_{i}},\phi_{5_{i}}\phi_{7_{i}}$) and the condition parameters (the first five parameters) of each one of the *M* rules ($|G_{r_{3_{k}}}\left|,\right|G_{r_{5_{k}}}\left|,\right|G_{r_{7_{k}}}|,\phi_{r_{5_{k}}},\phi_{r_{7_{k}}}$). For this comparison, the group of three gradient vectors represented by the condition parameters of that rule is rotated and reflected to minimize the Euclidean distance among the three pairs of gradient vectors.

To produce the distance between the two sets of gradient vectors (neighborhood and rule gradient vectors), the sum of the $L^{2}$ norms of the vector differences are calculated after rotating and reflecting the rule gradient vectors:

(7) $$d_{i_{k}}=∥G_{3_{i}}-G_{r_{3_{k}}}^{\prime }∥+∥G_{5_{i}}-G_{r_{5_{k}}}^{\prime }∥+∥G_{7_{i}}-G_{r_{7_{k}}}^{\prime }∥,$$

where $\left\{G_{3_{i}},G_{5_{i}},G_{7_{i}}\right\}$ correspond to the gradient vectors of the pixel and $\left\{G_{r_{3_{k}}}^{\prime },G_{r_{5_{k}}}^{\prime },G_{r_{7_{k}}}^{\prime }\right\}$ are the gradient vectors of rule *k* after undergoing a rotation of ϕ and a reflection if required (Figure 2b).

Thus, selecting a rule involves obtaining the minimum distance $d_{i_{k}}$ for each rule and then choosing the one, *s*, leading to the smallest distance value:

(8) $$s=argmin_{k\in \{1,2,\ldots ,M\}}d_{i_{k}}.$$

### 3.5. Updating the State of the CA

**Figure 3:**
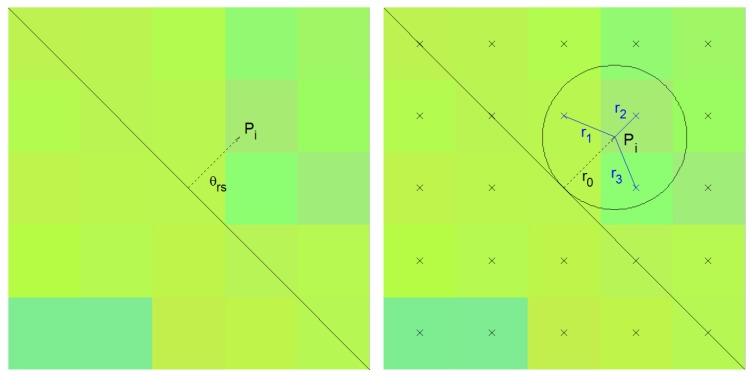
Selection of neighboring pixels for updating the state of cell *i*.

As indicated above, the last parameter of the rule vector represents the action that allows updating the state of cell *i*. This state is updated by modifying its representative spectrum through a weighted average with the spectrum of some of its neighbors. The neighbors that participate in the average are chosen by following the direction $\phi +\theta_{r_{{}_{s}}}$ for a distance of one pixel up to point $P_{i}$, (Figure 3). The spectra of the set of points located at a distance of no more than one pixel from $P_{i}$ will contribute to the weighted average together with the spectrum of *i*, leading to the updated state of cell *i*, $s_{i}^{\prime }$. Here, ϕ corresponds to the rotation angle of the rule, *s*, employed in the calculation of the distance $d_{i_{k}}$. $\theta_{r_{{}_{s}}}$, on the other hand, is the action parameter of rule *s*. Thus:

(9) $$\begin{array}{c} s_{i}^{\prime }=\sum_{j=1}^{n}w_{ij}\cdot s_{\mathrm{ij}}+w_{i}\cdot s_{i},\\ w_{ij}=\frac{f_{r}\left(r_{j}\right)}{\sum_{j=1}^{n}f_{r}\left(r_{j}\right)+f_{th}},\;w_{i}=\frac{f_{th}}{\sum_{j=1}^{n}f_{r}\left(r_{j}\right)+f_{th}},\\ f_{r}\left(r_{j}\right)=\left\{\begin{array}{cc} f_{th}, & \;\mathrm{if}\;\frac{1}{r_{j}}>f_{th},\\ \frac{1}{r_{j}}, & \;\mathrm{otherwise}, \end{array}\right. \end{array}$$

where $s_{i}^{\prime }$ denotes the updated spectrum and $s_{i}$ the original one for cell *i*; $s_{\mathrm{ij}}$ represents the spectrum of a neighboring cell *j*; *n* is the number of neighboring cells considered for the state update; $r_{j}$ is the distance between cell *j* and $P_{i}$; $w_{ij}$ corresponds to the weight for the spectrum of cell *j*; $w_{i}$ is the weight for the spectrum of cell *i* and, finally $f_{r}\left(r_{j}\right)$ represents a function that assigns weights depending on the distance between the points, $r_{j}$.

Following this procedure, which is summarized in Algorithm 1, the CA produces a new hyperspectral cube every iteration through its iterative application to the whole image. Therefore, if the transition rules are appropriately chosen, the final hyperspectral cube provided by the composition of the CA cell states (cell spectra) should be a segmented version of the image where each region comprises a narrow spectral range.

**Algorithm 1** MGCA**Input:***X*: image of *m* × *n* pixels and *N* bands **Output:**
*Y*: segmented image of *m* × *n* pixels and *N* bands
1:**for***p* →1 to *K* **do**  ▹ *K* iterations2:  **for** i → 1 to *n* · *m* **do**3:    Calculate $|G_{3_{i}}\left|,\right|G_{5_{i}}\left|,\right|G_{7_{i}}|,\phi_{3_{i}},\phi_{5_{i}},\phi_{7_{i}}$ using the spectral angle values, $\alpha_{ij}$4:    Select rule, *s*, by comparing the neighboring information $|G_{3_{i}}\left|,\right|G_{5_{i}}\left|,\right|G_{7_{i}}|,\phi_{3_{i}},\phi_{5_{i}},\phi_{7_{i}}$ with the first five parameters    of the transition rules $|G_{r_{3_{k}}}\left|,\right|G_{r_{5_{k}}}\left|,\right|G_{r_{7_{k}}}|,\phi_{r_{5_{k}}},\phi_{r_{7_{k}}}$    ▹ Image rotation and reflection are considered5:    Update the state of the cell *i* using the last parameter of the selected rule     ▹ A weighted spectral average of the spectrum of cell *i* and that of some of its neighbors is performed considering     all the spectral bands6:  **end for**7:**end for**


## 4. ECAS-II: Evolving the Cellular Automata

### 4.1. Introduction

ECAS, version I [58] and II [59,60], are evolutionary approaches for the generation of Cellular Automata (CA)-based segmentation algorithms. They provide the parameters or transition rules that govern their operation and behavior.

There are several reasons for choosing EAs as the method for the creation of the optimal transition rule set. First, EAs do not make strong assumptions regarding the underlying fitness landscape. Second, EAs work on a population of solutions instead of a single point. This makes them less likely to be misled by local optima when searching for the optimum set of transition rules.

As indicated, EAs operate over a population of encoded possible solutions to the problem. A new population of possible solutions is generated every generation by means of the selection and variation of the members of the population as a function of their evaluation according to a fitness function. This process is iterated until an individual with sufficient fitness (candidate solution) is found or when a previously selected computational limit is reached.

### 4.2. Differential Evolution

In particular, the evolutionary algorithm chosen in this implementation of ECAS-II to create the set of transition rules governing CA behavior has been a Differential Evolution Algorithm (DE) [61]. DE has proven to work efficiently for minimizing non-continuous, noisy, non-differentiable, nonlinear, flat objective functions, even when they are multi-dimensional or present many local minima.

The most important components of the DE algorithm are:

- Representation (Definition of the Individuals): Each CA that makes up the DE population is encoded as a D=6·M floating point value vector. These values are a direct representation of the parameters shown in Equation (6). The values corresponding to $|G_{r_{3_{k}}}\left|,\right|G_{r_{5_{k}}}|$, and $|G_{r_{7_{k}}}|$ belong to the $\left[0,\sqrt{2}\right]$ interval and those corresponding to $\phi_{r_{5_{k}}},\phi_{r_{7_{k}}}$ and $\theta_{r_{k}}$ are in the [0,2π] interval.
- Population: NP D-dimensional parameter vectors $x_{i,G},i=1,2,\ldots ,NP$ constitute the population considered in each generation *G*. The initial vector population is chosen randomly and spread out over the entire parameter space.
- Variation Operators: Mutation
  New individuals are generated in DE through the addition of the weighted difference of two vectors (individuals) of the population to a third one. For each parameter vector, $x_{i,G},i=1,2,\ldots ,NP$, a mutated individual is created as: $v_{i,G+1}=x_{r_{1},G}+F\cdot \left(x_{r_{2},G}-x_{r_{3},G}\right)$ with random, integer, mutually different indexes $r_{1},r_{2},r_{3}\in \left\{1,2,\ldots ,NP\right\}$ and where F>0. *F* is a real constant ∈[0,2] controlling the differential variation gain $\left(x_{r_{2},G}-x_{r_{3},G}\right)$.
- Variation Operators: Crossover
  With the objective of increasing diversity in the new population, a crossover operator is proposed that constructs new individuals $u_{i,G+1}=\left(u_{1i,G+1},u_{2i,G+1},\ldots ,u_{Di,G+1}\right)$ as:
  $$\begin{array}{cccc} u & {}_{ji,G+1}= & \left\{\begin{array}{cc} v_{ji,G+1} & \mathrm{if}\;(randb(j)\leq CR)\;\mathrm{or}\;j=rnbr(i),\\ x_{ji,G} & \mathrm{if}\;(randb(j)>CR)\;\mathrm{and}\;j\neq rnbr(i), \end{array}\right. & j=1,2,\ldots ,D, \end{array}$$
  where randb(j) is the jth result from a uniform random number generator with outcomes ∈[0,1]. CR is the crossover parameter ∈[0,1], provided by the user; rnbr(i) is a random value ∈1,2,…,D to ensure that at least one parameter from $v_{i,G+1}$ is sent to $u_{i,G+1}$.
- Selection: To decide if a potential parameter vector should be included in generation G+1, $u_{ji,G+1}$ is compared to $x_{i,G}$. If the fitness value of $u_{ji,G+1}$ is smaller than $x_{i,G}$, then $x_{i,G+1}$ is set to $u_{ji,G+1}$; otherwise, the old value $x_{i,G}$ is retained. More details of this DE implementation can be found in [61].

The key to the versatility of ECAS-II lies in how the individuals are evaluated and how the training image set is created. This is presented in the following two subsections.

### 4.3. Evaluation of Individuals and Fitness Function

In order to evaluate each individual (each CA and here each MGCA), or calculate its fitness, we have chosen to determine how apt it is at segmenting a set of images that are provided together with the desired segmentation (ground truth). These images will be considered the training set and, as mentioned later, should reflect the segmentation level desired by the user. To evaluate a CA, it is applied to the training image and its state is compared to the ground truth after a set number of iterations. Different images are used for the evaluation of each individual in order to improve generalization and prevent circularity. These images can come from a pre-generated collection, or they can be generated online.

The cost function to be minimized in ECAS-II is related to the maximum value of two error measurements: intra-class ($e_{intra}$) error and inter-class ($e_{inter}$) error:

(10) $$e=max(e_{intra},e_{inter}).$$

**Figure 4:**
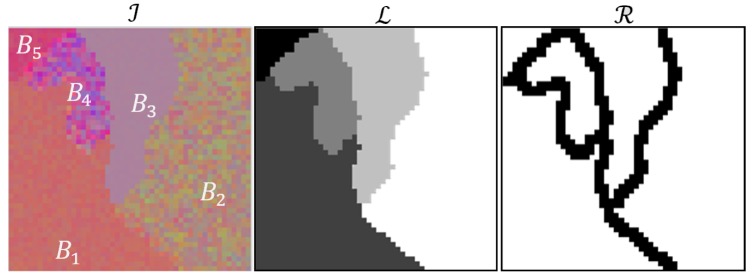
Synthetic RGB image, ground truth and edge image.

In order to make the description of these error measurements clearer, we will introduce some notation. First of all, let $I={\left\{x_{p}\right\}}_{p=1}^{P\times Q}$ be a training image of size P×Q, where $x_{p}$ is an *N*-dimensional vector. Let $L{=\left\{l_{p}\right\}}_{p=1}^{P\times Q}$ be the ground truth of I with $\;l_{p}\in S$, being $S=\{1,\;2,\ldots ,s_{k},\;\ldots ,M\}$ the set of labels in L. Let $R={\left\{r_{p}\right\}}_{p=1}^{P\times Q}$ with $r_{p}\in \left\{0,1\right\}$ be a binary image that defines every pixel L as a border {0}, or an interior {1} pixel,. I can also be taken as the merging of regions associated with the same label,$\;I={⋃}_{k=1}^{M}B_{k}$, where $B_{k}=\{x_{p}\;\left|\;l_{p}=s_{k}\}\right.$. Using the labeling in L, $B_{k}\;$ includes two subsets, one containing only interior pixels and another one containing border pixels. Therefore, $B_{k}=I_{k}\cup \;F_{k}$, where $I_{k}=\{x_{p}\;\left|\;l_{p}=s_{k}\;\wedge {\;r}_{p}=1\}\right.$ and $F_{k}=\{x_{p}\;\left|\;l_{p}=s_{k}\;\wedge {\;r}_{p}=0\}\right.$. $F_{k}$ can be divided into M−1 subsets, $F_{k}={⋃}_{k^{\prime }=1}^{M}F_{kk^{\prime }}^{\prime }$ with $k^{\prime }\neq k$, with $F_{kk^{\prime }}^{\prime }$ comprising the pixels located in the borders between $B_{k}$ and $B_{k^{\prime }}$ (Figure 4).

Now, taking into account this notation, two types of errors may be defined. An intra-class error indicates how homogeneous the different regions of the image are. A region is defined as an area of the ground truth of the training image whose pixels display the same label. Calculation of this error consists of obtaining the maximum value of two associated error measures: the local intra-region error ($e_{local-intra}$) and the non-local intra-region error ($e_{nonlocal-intra}$):

(11) $$e_{intra}=\max\left(e_{local-intra},\;e_{nonlocal-intra}\right)\;,$$

with the local intra-region error being:

(12) $$e_{local-intra}=\frac{\sum_{k=1}^{M}\left[\sum_{x_{p}\in I_{k}}\left(\sum_{j=1}^{8}\frac{\alpha_{pj}}{8}\right)\right]}{\sum_{k=1}^{M}\#I_{k}},$$

where $\alpha_{pj}$ is the normalized spectral angle when comparing a given pixel $x_{p}$ to its neighbor $x_{j}$. $\#I_{k}$ denotes the cardinality of subset $I_{k}$. This $e_{local-intra}$ error provides an estimation of the spectral similarity of neighboring pixels that display the same label in L. This similarity or local homogeneity is defined for each $I_{k}$ as:

(13) $$H_{k}=\frac{\sum_{x_{p}\in I_{k}}\left(\sum_{j=1}^{8}\frac{\alpha_{pj}}{8}\right)}{\#I_{k}}.$$

Conversely, in order to define $e_{nonlocal-intra}$, *V* pixel pairs are randomly selected for each $I_{k}$ subset, ${\{x}_{kv},y_{kv}\}\;\in I_{k}\;$ with $\;v=\left\{1,2,\ldots V\right\}$. $e_{nonlocal-intra}$ can then be expressed as:

(14) $$e_{nonlocal-intra}=\frac{\sum_{k=1}^{M}\left[\sum_{v=1}^{V}\frac{\alpha_{kv}}{V}\right]\#I_{k}}{\sum_{k=1}^{M}\#I_{k}},$$

where $\alpha_{kv}\;$ is the normalized spectral angle obtained from $x_{kv}$ and $y_{kv}$. Thus, $e_{nonlocal-intra}$ provides a measure of how spectrally homogeneous are the pixels, located in the same region, but not necessarily neighbors.

Finally, the inter-region error measure, $e_{inter},\;$ represents an estimation of how dissimilar the different regions in the segmented image are. To define $e_{inter}$, a set of *U* pairs of pixels are randomly selected for each $F_{kk^{\prime }}^{\prime }\cup F_{k^{\prime }k}^{\prime }$, ${\{x}_{ku},y_{k^{\prime }u}\}$, with $x_{ku}\in {F^{\prime }}_{kk^{\prime }}$$x_{k^{\prime }u}\in {F^{\prime }}_{k^{\prime }k}$, and $v=\left\{1,2,\ldots V\right\}$.

(15) $$e_{inter}=\frac{\sum_{k=1}^{M}\left[\sum_{k^{\prime }=1}^{M}\frac{\sum_{u=1}^{U}f\left(\alpha_{kk^{\prime }u},\;\;\;H_{k},\;\;\;H_{k^{\prime }}\right){\#B}_{k^{\prime }}\;}{U\sum_{k^{\prime }=1}^{M}{\#B}_{k^{\prime }}}\right]{\#B}_{k}}{\sum_{k=1}^{M}{\#B}_{k}},$$

(16) $$f\left(\alpha_{kk^{\prime }u},\;H_{k},\;H_{k^{\prime }}\right)=\left\{\begin{array}{ccc} 1, & if & \alpha_{kk^{\prime }u}\leq \;\;\;H_{k}+\;H_{k^{\prime }},\\ 0, & if & otherwise, \end{array}\right.$$

where $\;{\;\alpha }_{kk^{\prime }u}$ is the normalized spectral angle in the case of $x_{ku}$ and $y_{k^{\prime }u}$.

## 5. ECAS-II: Creating the Training Image Dataset

**Figure 5:**
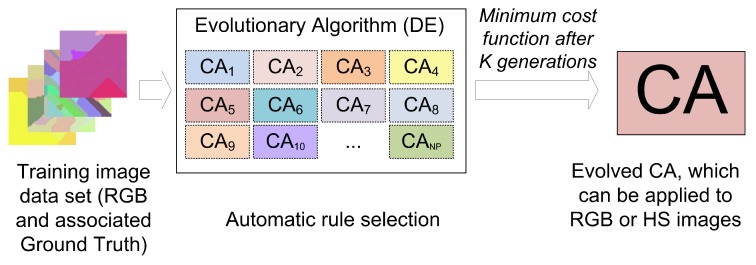
Diagram of the ECAS-II training process.

**Figure 6:**
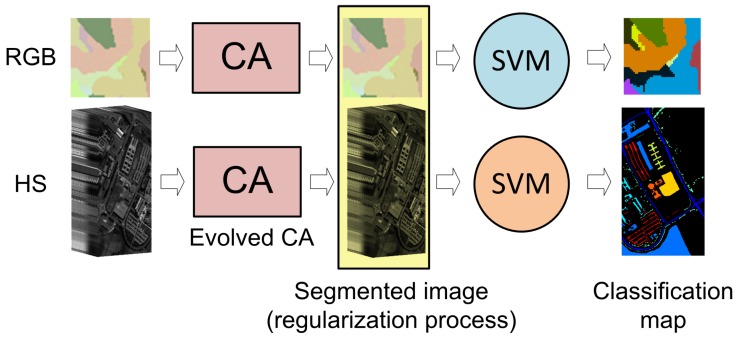
Diagram of the MGCA + SVM classification process.

As a first approximation, it may seem advantageous to select real hyperspectral images as training images for optimizing the transition rules of the CA in the evolutionary process. However, what happens in practice is that publicly available hyperspectral image sets are not common and the number of images they contain is very limited, forcing us to perform the training process always using the same images. Furthermore, the reliability of the labeling of the available hyperspectral images is often questionable, especially in borders between areas, leading to skewed evaluations of the CAs. This makes a good case for making use of synthetic datasets that reflect the segmentation the user requires. Furthermore, as the spectral distance measure used in ECAS-II is the spectral angle, this makes the algorithm independent of the dimensionality of the image over which it is applied. This can be exploited to speed up and simplify the evolutionary process. In this case, to make the generation of training sets more straightforward, we propose using low dimensional (RGB) training images during the evolutionary process. This way, computational complexity is reduced and, as long as the Spectral Angle behavior is preserved, the CA resulting from ECAS-II should be valid for any dimensionality. Schematic diagrams of how ECAS-II and the MCGA+SVM classification process work are displayed in Figure 5 and Figure 6).

The use of synthetic RGB images as the training set for the DE algorithm entails the following benefits:

- Each synthetic RGB image is created with an associated fully reliable ground truth. This guarantees that the cost function output only depends on the evaluated CA.
- The creation of synthetic RGB images may be made completely automatic. This allows for evaluating each individual with a different image created online, or by previously creating a large collection of synthetic RGB images and just picking one of them randomly at run-time.
- The controlled creation of synthetic RGB images together with appropriate ground truth values makes it feasible to train the CA in order to achieve any desired specialized segmentation algorithm.

With a view to adapt the behavior of the segmenting CA to the required type of segmentation in terms of grain or detail, it is necessary to control and adjust the features of the RGB images used as a training set in the evolutionary algorithm and their associated ground truths. To this end, we have developed a new tool (The tools used in this work for the creation of synthetic RGB images and the estimation of the image feature parameters from real hyperspectral images are available through downloadable MATLAB code at http://gii.udc.es/gii_hyperspectral_repository) that creates synthetic RGB images and their corresponding ground truths based on four image feature parameters that characterize their spectral and spatial properties:

- $r_{max}$: Related to the averaged intra-class spectral distances between neighboring pixels,
- $\left[s_{min}s_{max}\right]$: Minimum and maximum values related to averaged inter-class spectral distances,
- *N*: Related to the density of different classes in the ground truth,
- $D_{max}$: Related to the ruggedness of the borders that delimit different classes.

**Figure 7:**
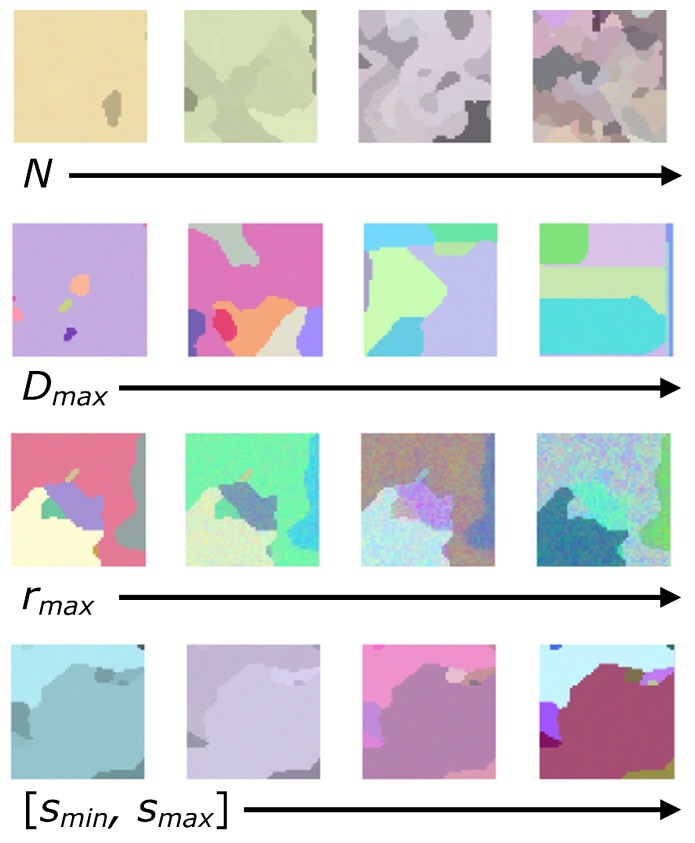
Synthetic RGB images created with the proposed tool. The influence of $N,D_{max},r_{max}$ and $\left[s_{min},s_{max}\right]$ over the geometric and spectral aspects of the images can be appreciated.

Figure 7 depicts how these parameters affect the geometric and spectral features of synthetic RGB images. Thus, depending on the type of segmentation one is looking for, different values for these parameters can be chosen in order to create the training set. More details on this image feature parameterization can be found in [62].

Nevertheless, sometimes choosing the appropriate parameters for the type of segmentation a user requires may not be obvious. To solve this problem, a second auxiliary tool has been developed. This tool is provided with a very small number of images and their ground truths that correspond to the type of segmentation the user desires, and it automatically estimates the appropriate values for $N,D_{max},r_{max}$ and $\left[s_{min},s_{max}\right]$.

Summarizing, in the proposed methodology, ECAS-II can modulate the MGCA transition rules depending on the features of the synthetic training images that are created, which have been constructed to represent the type of hyperspectral images that will be processed and the characteristics of the segmentation that is required.

## 6. Classification Step Using SVM

Comparing the results of segmentation algorithms usually implies performing a posterior classification process, that is, a labeling process over the segmented regions, as this is how the results are usually presented in the literature. In the case of the approach presented here, to produce a final classification image from the segmented data, any pixel-wise classifier can be used. In the examples presented in this paper, we have applied a direct SVM based pixel-wise classification to the ECAS-II generated MGCA segmentation results (Figure 6). This step is carried out using a multi-class pairwise (one versus one) SVM classification with the specific implementation found in [63]. For all the experiments, a Gaussian Radial Basis Function (RBF) kernel is used, and the parameters *C* and γ were determined by five-fold cross-validation.

## 7. Application of ECAS-II Generated MGCAs + SVM to Hyperspectral Images

To test the performance of the ECAS-II generated MGCA based segmentation algorithms, the whole strategy was first applied to synthetic hyperspectral images to clearly demonstrate its validity in well-controlled images. As a second step, the strategy was then applied to well known real benchmark hyperspectral images and to recently acquired real hyperspectral images extracted from a new online public repository to compare its results to those of other algorithms found in the literature.

This section is organized as follows: in Section 7.1, the ECAS-II generated MGCA (for simplicity, we will refer to it as just ECAS-II ) + SVM method is applied to synthetic hyperspectral images. This part is devoted to the demonstration of the impact of applying the ECAS-II method prior to the classification step over perfectly labeled multidimensional images. Section 7.2 is focused on comparing the classification performance of the multistage ECAS-II + SVM algorithm to that of other existing techniques when it is applied to real benchmark hyperspectral images. Finally, in Section 7.3, the proposed algorithm is tested using real hyperspectral images from the GII-HSEG repository in order to consider images with spatial and spectral characteristics different than those usually considered in the literature.

The quality of the classification results was computed using the following commonly used quantitative metrics:

1. Overall Accuracy (OA): percentage of correctly classified pixels.
2. Average Accuracy (AA): mean of the percentage of correctly classified pixels for each class.
3. Kappa coefficient (κ): percentage of agreement (correctly classified pixels) corrected by the number of agreements that would be expected purely by chance.
4. Class-specific accuracy: percentage of agreement of each class.

### 7.1. Application to Synthetic Hyperspectral Images

This section is devoted to quantitative analysis in order to demonstrate the capabilities of the ECAS-II method, when applied to perfectly labeled and controlled multidimensional images. For this purpose, several images have been created synthetically. These test images were created starting from five 64-band base spectra, which were corrupted by noise, spectral mixtures and artifacts.

**Figure 8:**
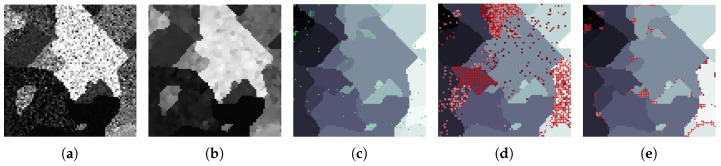
(**a**) 13th band of a very noisy synthetic 64-band image. (**b**) 13th band of the ECAS-II output, (**c**) Ground truth (green points represent training samples), (**d**) Classification produced by the SVM alone (red points represent misclassified pixels), (**e**) Classification produced by ECAS-II + SVM.

**Figure 9:**
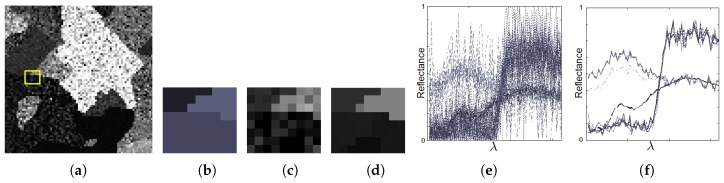
(**a**) 13th band of a very noisy synthetic 64-band image, (**b**) Detail (marked with a yellow square in Figure 9a) of the associated ground truth, (**c**) 13th band of the detailed area, (**d**) 13th band of the ECAS-II output over the detailed area, (**e**) Spectra of pixels shown in Figure 9c, (**f**) Spectra of pixels shown in Figure 9d.

Figure 8a shows the 13th band of a synthetic image in which the pixels have been corrupted by a high level of random noise. An MGCA obtained using the ECAS-II algorithm is applied for 10 iterations to the noisy synthetic image and the resulting 13th band is shown in Figure 8b. The CA that was applied in this case was evolved using the parameters shown in Table 1. The SVM method has been trained using an extremely low number of reference samples, approximately 1 percent of each class. These training samples are marked with green dots in Figure 8c. Additionally, Figure 8d shows the classification map provided by the SVM method applied to the original synthetic image, and Figure 8e the classification map when the SVM is applied to the segmented output provided by the CA. For both cases, misclassified pixels are marked with red dots. Figure 9 displays the detail corresponding to a small area of the image and includes a representation of the spectra of the pixels. Table 2 shows the improvement in terms of accuracy metrics introduced by the CA stage over the classification results using the raw SVM method.

**Figure 10:**
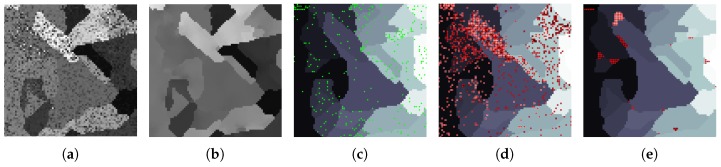
(**a**) 13th band of a highly spectrally mixed synthetic 64-band image. (**b**) 13th band of the ECAS-II output (**c**) Ground truth (green points represent training samples), (**d**) Classification produced using the SVM by itself (red points represent misclassified pixels), (**e**) Classification produced by ECAS-II + SVM.

**Figure 11:**
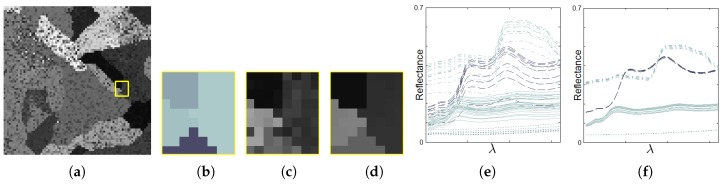
(**a**) 13th band of a highly spectrally mixed synthetic 64-band image, (**b**) Detail (marked with a yellow square in Figure 11a) of the associated ground truth, (**c**) 13th band of the detailed area, (**d**) 13th band of the CA output over the detailed area, (**e**) Spectra of pixels shown in Figure 11c, (**f**) Spectra of pixels shown in Figure 11d.

For the next experimental test, instead of adding random noise to corrupt the pixel spectra, we have created a synthetic 64-band image in which several spectra from a spectral database have been mixed to conform each pixel’s spectrum. This situation is much more common, especially in remote sensing images, as it corresponds to endmember mixtures in pixels. The visual results for this test are presented in Figure 10 and the accuracies obtained for the classification are indicated in Table 2. The best values are shown in bold typeface. To improve the understanding of the operation of the CA over this synthetic image, Figure 11 depicts the effect of applying the CA over the spectra of pixels from a detailed area in which four different regions intersect. Figure 11e displays the original spectra before the application of the CA. Figure 11f represents the pixel spectra after 35 iterations of the CA. Figure 11f shows how the CA gradually modifies the pixel spectra. These final pixel spectra converge to some spectral clusters. As a consequence of this, as reflected in Figure 11 and Table 2, the application of the CAs obtained using the ECAS-II algorithm simplifies the posterior classification stage and makes it more effective.

The results of this section validate the potential of the proposed methodology. These CAs were produced using simple synthetic RGB image datasets for the training step of the ECAS-II method and produce structures that when directly applied to multi-dimensional images result in satisfactory segmentations considering hyperspectral images with different spatio-spectral characteristics.

### 7.2. Application to Real Benchmark Hyperspectral Images

After testing the segmentation capabilities of the ECAS-II generated MGCAs, this section aims to verify their behavior in terms of segmentation over real images and seeks to compare the performance of the proposed approach to that of other methods found in the literature. To this end, three benchmark hyperspectral scenes were considered. The first hyperspectral image is the Pavia University scene, which was recorded by the ROSIS-03 satellite sensor. This 610×340 pixel image includes 115 spectral bands. The second one is from the AVIRIS sensor over Salinas Valley, CA, USA. It is a 512×217 pixel image with 224 spectral bands. Finally, the third image we have used corresponds to the well-known 145×145 pixel and 200-band Indian Pines (Indiana) image recorded by the AVIRIS sensor.

In order to produce segmenters for each scene using the ECAS-II algorithm, three different MGCAs were trained using synthetic RGB images with similar feature parameters to those of the corresponding hyperspectral images. All the CAs were evolved using the parameters shown in Table 1. The process involved first normalizing the hyperspectral images in terms of intensity and then applying the SVM. The SVM parameters (*C* and γ) used, obtained by means of a five-fold cross validation procedure, are also indicated in Table 3.

The class-specific accuracies for the ECAS-II + SVM and the raw SVM algorithms using in the training procedure 25 samples of each class for the Pavia, Indiana and Salinas scenes, respectively, are presented in Table 3 and the best results are shown in bold typeface. The number of samples per class for the training process will also be limited to 80% of the samples in each class. The quantitative results show accuracy improvements for most of the classes. In some cases, as for the shadows class in the Pavia image, the accuracy is degraded by the application of ECAS-II. However, the classification of shadow classes is very controversial. They do not represent a particular material (or spectral signature) by themselves as the class may include many different spectra. This class is just a representation of pixels with lower spectral intensity than the regular pixels in their corresponding classes.

Next, to quantitatively compare the classification results obtained for these scenes to other approaches, a total of six methods were extracted from the literature: two pixel-wise classification techniques that do not include spatial correlations in their processing (SVM and MLR [19]); two methods that use local relationships among first-order neighboring pixels to correct spectral and spatial distortions as a preprocessing step of a MLR model (ppMLR) [19], reducing noise and improving the class separability in the spectral domain, and, as a postprocessing approach, working on the label image or class probabilities obtained from a MLR classifier (MLRpr) [19]; and a collaborative representation-based nearest neighbor (CRNN) algorithm [15], where the CR is computed by an l2-norm minimization with a Tikhonov regularization matrix.

**Figure 12:**
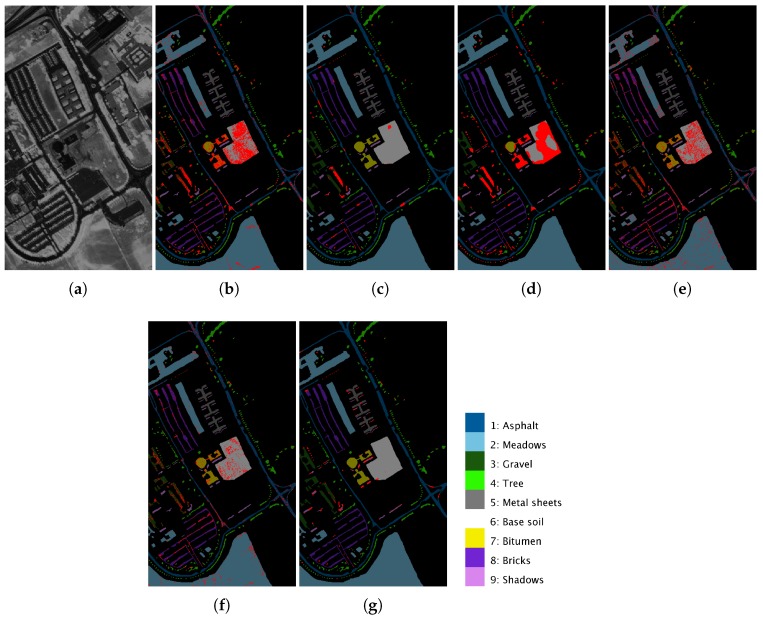
2D angular transformation of the original hyperspectral image (**a**) and the results of the application of MLR (**b**); ppMLR (**c**); MLRpr (**d**); CRNN (**e**); SVM (**f**); and ECAS-II-SVM (**g**) classification methods to the Pavia scene. Misclassified pixels are marked with red dots. The classification methods have been trained using 3% of the samples of each class.

**Figure 13:**
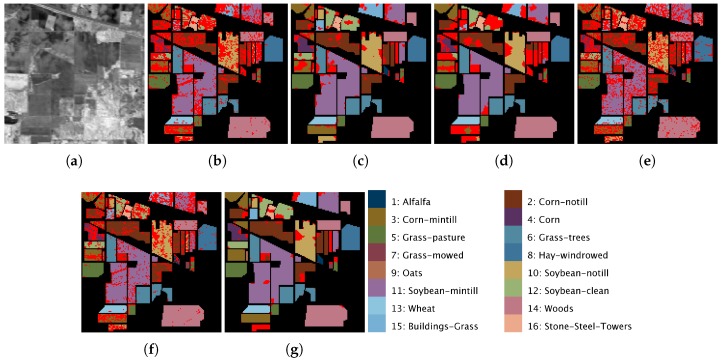
2D angular transformation of the original hyperspectral image (**a**) and the results of the application of MLR (**b**); ppMLR (**c**); MLRpr (**d**); CRNN (**e**); SVM (**f**); and ECAS-II-SVM (**g**) classification methods to the Indiana scene. Misclassified pixels are marked with red dots. The classification methods have been trained using 3% of the samples of each class.

**Figure 14:**
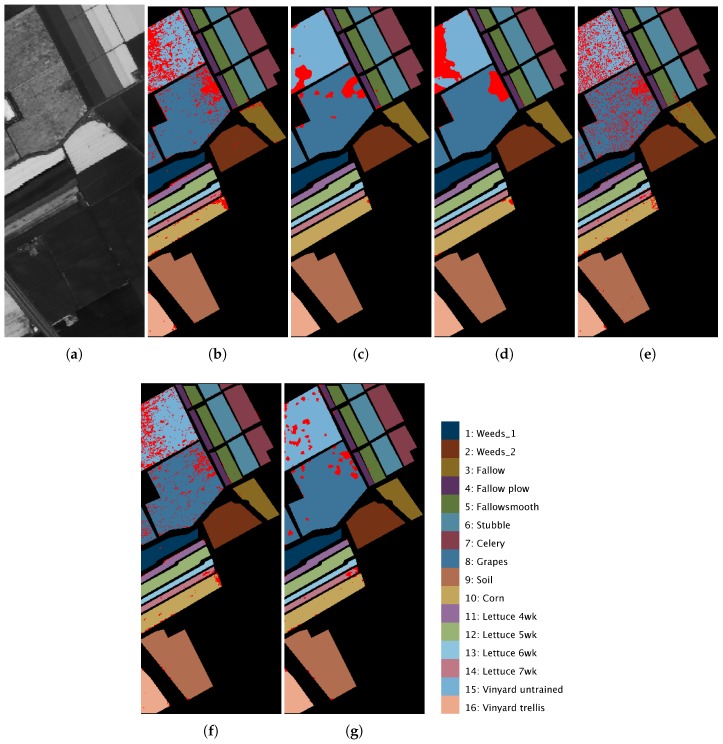
2D angular transformation of the original hyperspectral image (**a**) and the results of the application of MLR (**b**); ppMLR (**c**); MLRpr (**d**); CRNN (**e**); SVM (**f**); and ECAS-II-SVM (**g**) classification methods to the Salinas scene. Misclassified pixels are marked with red dots. The classification methods have been trained using 10% of the samples of each class.

Figure 12a, Figure 13a and Figure 14a show a 2D angular transformation of each original hyperspectral image. In these images, the value of each pixel corresponds to the spectral angle between its spectrum and a reference one with all of its bands saturated. Figure 12b–g, Figure 13b–g and Figure 14b–g represent the final classification results for the MLR, ppMLR, MLRpr, CRNN, SVM and ECASII-SVM algorithms when the algorithm was trained using 3% of the samples of each class for the Pavia, Indiana and Salinas scenes. In these figures, we have indicated the pixels that were misclassified using red dots. It can be clearly appreciated that the classification methods that introduce contextual (spatial) information in the processing of the images result in lower classification errors. In particular, the ECASII-SVM is the method with the best performance for these images. Moreover, these visual results reveal that the application of the ECAS-II MGCA prior to the SVM algorithm leads to a reduction of the misclassified pixels and that errors tend to take place in concentrated areas.

**Figure 15:**
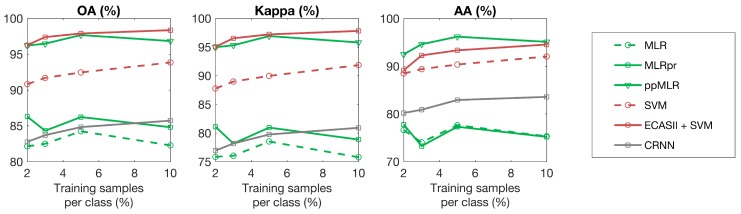
Classification accuracies (%) for different algorithms applied to the Pavia scene using different numbers of training samples.

**Figure 16:**
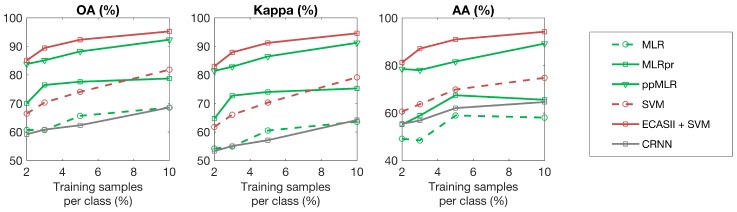
Classification accuracies (%) for different algorithms applied to the Indiana scene using different numbers of training samples.

**Figure 17:**
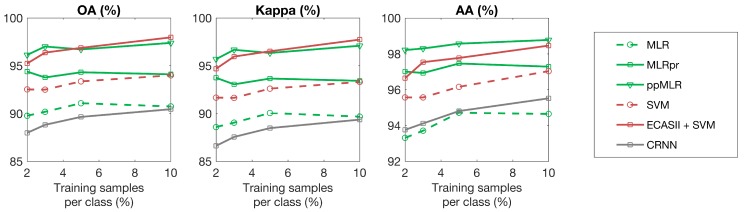
Classification accuracies (%) for different algorithms applied to the Salinas scene using different numbers of training samples.

For each hyperspectral scene, the classification algorithms under consideration were trained using different percentages of samples for each class. The results with respect to overall accuracy, average accuracy and kappa coefficient are plotted in Figure 15, Figure 16 and Figure 17 showing that ECAS-II CAs followed by an SVM pixel-wise classification stage provide very competitive results when compared to the selected state-of-the-art classification algorithms.

Finally, it is important to note that one of the most interesting features of Evolutionary and CA-based approaches is that their concurrent calculation is straightforward. Therefore, implementations can be provided that can achieve very good processing times over GPUs or other distributed processing platforms. To provide some numbers on performance, as an indication of computational performance for this approach, we have tested the algorithm both on a CPU based system using OpenMP and a GPU based system with shared memory using CUDA. In the first case, the algorithm was run on a quad-core Intel Core i5-3470 PC at 3.20 GHz with 8 GB of RAM using gcc 4.8.4 with OpenMP 3.0 support under Linux 14.04. The execution times in seconds were 206.57 for the Pavia image, 29.69 for the Indiana scene and 275.16 for the Salinas image. In the second case, we made use of a Kepler NVIDIA GeForce GTX Titan with 14 SMXs and 192 CUDA cores each. The execution times here were much better, achieving 13.31 s for the Pavia image, 3.21 for the Indiana scene and 15.72 for the Salinas image.

### 7.3. Application to Real Hyperspectral Images from the GII-HSEG Repository

This last experimental subsection has been included here to validate ECAS-II over hyperspectral images with different spatial and spectral parameter descriptors to those of the Salinas, Pavia and Indiana scenes. This would ensure that the ECAS-II strategy is valid independently of the type of hyperspectral images to which it is applied.

**Figure 18:**
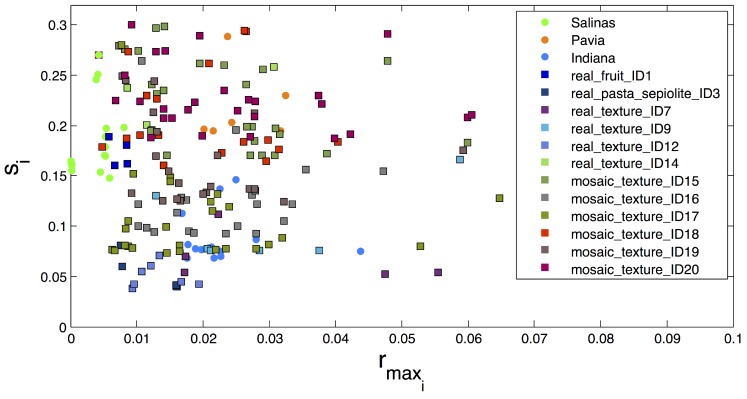
Averaged inter-class distance (*y*-axis) between each class and the rest of the classes vs. averaged intra-class distance (*x*-axis) of each class in terms of normalized spectral angle between 0 and 1 for the Pavia, Indiana, and Salinas scenes and selected images from the GII-HSEG repository.

**Figure 19:**
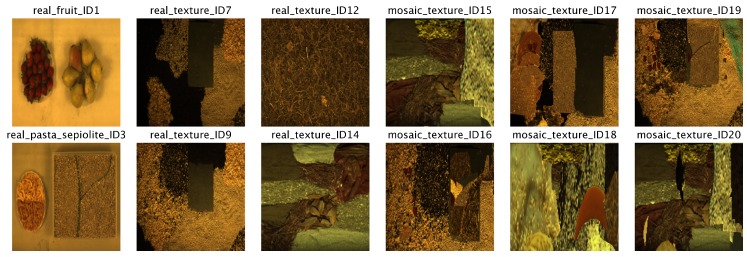
RGB false-color composition of hyperspectral images used in Figure 18.

**Figure 20:**
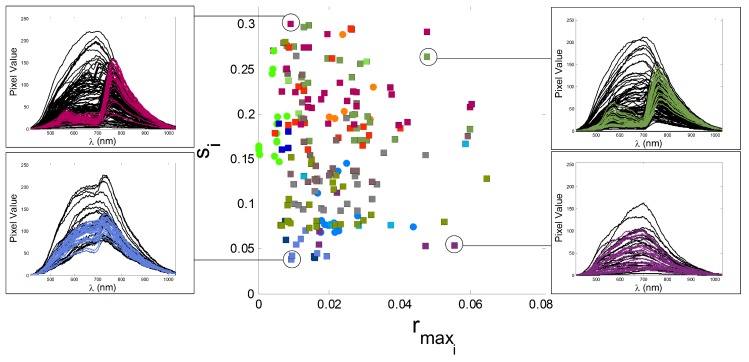
Spectra of pixels from the *real_texture_ID7*, *real_texture_ID12*, *mosaic_texture_ID15* and *mosaic_texture_ID20* scenes. For each graph, spectra of randomly selected pixels belonging to certain classes from the corresponding scene are plotted in color following the legend of Figure 18, while spectra from the remaining classes for the same scene are plotted in black.

The images from the GII-HSEG repository (Real hyperspectral images from the GII-HSEG repository are available at http://gii.udc.es/gii_hyperspectral_repository) cover different types of scenarios: isolated low-textured objects on a light background, isolated highly-textured regions on a light background, adjacent highly-textured regions without a common background and objects half-buried under materials of great heterogeneity and profuse texture. Figure 18 graphically shows the spectral variations between different scenes, by representing the image feature parameters in terms of the averaged intra-class distance of each class vs. the averaged inter-class distance between each class and the rest of the classes in terms of normalized spectral angle (between 0 and 1) for the Pavia, Indiana, Salinas scenes and some images from the GII-HSEG repository (Figure 19). In this graph, it is easy to see that the hyperspectral image labeled as *real_texture_ID7* exhibits high intra-class distances ($r_{{max}_{i}}$) keeping the inter-class distances ($s_{i}$) low. On the other hand, image *real_fruit_ID1* presents low spectral distances between pixels belonging to the same class, but the spectral distance between pixels from different classes is quite high. The idea here was to select images aiming to complement the spectral parameter space. It can be noticed that there are no points in the spectral parameter space for which $r_{{max}_{i}}>s_{i}$. This is because of the difficulty of setting up a scene in which pixels belonging to the same class are more spectrally heterogeneous than the spectral differences between pixels from distinct classes. For a better grasp of Figure 18, in Figure 20, the spectra of some pixels belonging to the *real_texture_ID7*, *real_texture_ID12*, *mosaic_texture_ID15* and *mosaic_texture_ID20* scenes are shown. For each graph, the spectra of randomly selected pixels belonging to a certain class from the corresponding scene are plotted in color following the legend of Figure 18, while the spectra from the remaining classes for the same scene are plotted in black. It is worth noting that, for example, in the case of the *real_texture_ID7* scene, the spectral distances between pixels belonging to the same selected class are almost the same as the ones with respect to pixels from the remaining classes, which is in line with a high value of $r_{max_{i}}$ and a low value of $s_{i}$.

From these twelve hyperspectral scenes, we have selected five images in order to assess the validity of the ECAS-II method. These images have been classified using an SVM algorithm with the specific *C* and γ parameters shown in Table 4, which were selected through a five-fold cross-validation procedure. The SVM algorithm has been applied pixel-wise to the original data cube and the segmented image provided by the ECAS-II CA. The SVM method has been trained and applied 10 times using different training samples. The averaged accuracy performances obtained are shown in Table 4.

In this section, the segmentation step has been carried out using three different evolved MGCAs, the first one to segment image *real_fruit_ID1*, the second one to segment image *real_pasta_sepiolite_ID3* and the third one to segment images *real_texture_ID7*, *real_texture_ID9* and *real_texture_ID12*.

**Figure 21:**
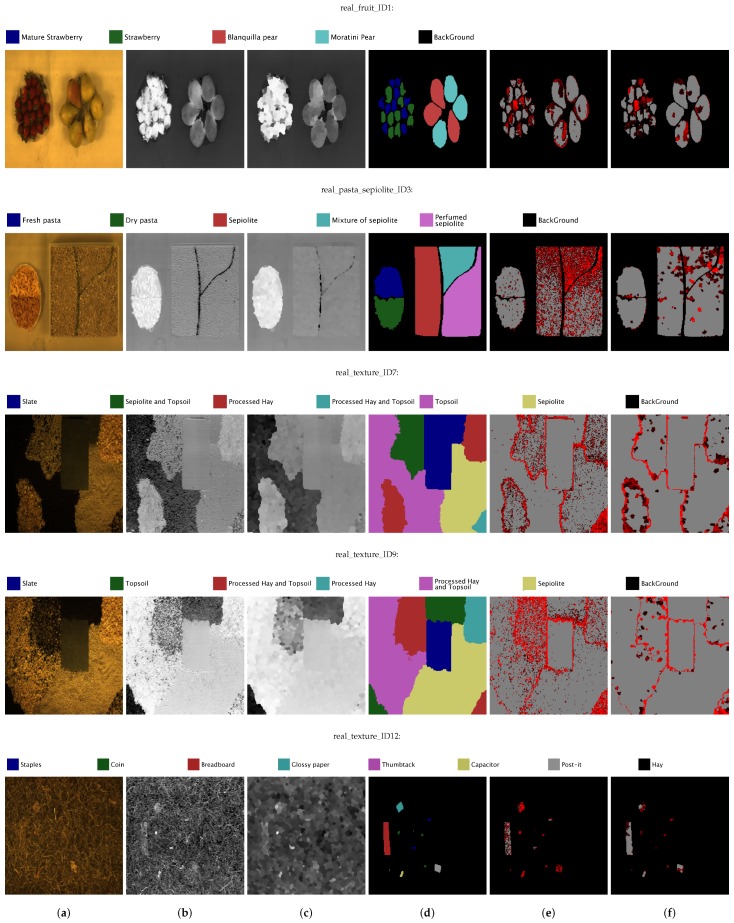
RGB false-color composition (**a**); 2D transformation of the original scene (**b**) and of the segmented image provided by ECAS-II (**c**); ground truth (**d**); SVM based pixel classification applied to the original scene (**e**); and to the segmented image provided by ECAS-II (**f**) showing only the labeled areas and marking misclassified pixels with red dots (a brighter red pixel means that it has been misclassified more times after applying the SVM algorithm 10 times).

Visual results for all the images are displayed in Figure 21. The images of Figure 21a show the RGB false-color composition for each scene. Figure 21b,c depict a 2D transformation of the original scenes and the segmentation results provided by ECAS-II. The ground truths are presented in Figure 21c and their legends are displayed above the row corresponding to each scene. Figure 21e,f represent the SVM based classification applied to both the original scene and the segmented one provided by ECAS-II. In these last images, only the labeled areas are colored in gray and misclassified pixels are colored in red of varying intensity (a brighter red pixel means that it has been misclassified more times when the SVM algorithm has been repeatedly applied 10 times).

All of the SVM results applied to the original or segmented scenes demonstrate that the selected use cases (discrimination between types of fruits, degree of ripeness, etc.) are appropriate to benefit from hyperspectral technology. The improvement that the ECAS-II segmentation step introduces in the accuracy performances depends on the spatial and spectral characteristics of the scene. When the regions exhibit high levels of spatio-spectral textures, the inclusion of spatial information is highly relevant in order to obtain better classification performances. This is more noticeable in the *real_pasta_sepiolite_ID3* and *real_texture_ID12* images, where the increase in the accuracy measurements is Δκ=20.13%, ΔOA=14.97%, ΔAA=12.68% and Δκ=32.33%, ΔOA=19.07%, ΔAA=24.61%, respectively. In the *real_pasta_sepiolite_ID3* image, the difference between sepiolite and perfumed sepiolite classes is an added purple-colored component of much lower granularity that gives off the smell of the perfumed sepiolite and is responsible for the salt-and-pepper noise in the classification result of directly applying the SVM to the original scene. In image *real_texture_ID12*, the fact that objects are half-buried poses a further difficulty for the classification task, and the reason for the worst result in terms of accuracy performances for the SVM applied to the original image. However, the inclusion of spatial information by means of the ECAS-II segmentation step significantly improves the classification accuracies for these cases.

## 8. Conclusions

This paper has addressed a series of issues present in hyperspectral, or generally multidimensional, image segmentation processes, such as the lack of appropriately labeled reference images, the user/application dependence of the required segmentation, or the use of all the information provided by the high dimensionality of the images. To this end, the paper describes a strategy involving a specifically designed dimension independent Cellular Automata based structure we have called Multi-Gradient based Cellular Automata (MGCA) and an evolutionary approach, called ECAS-II, that is able to generate the appropriate transition rules for MGCAs from low dimensional synthetic training images. These images are created online from the parametrization of certain characteristics of the real images to be processed. The combined approach has been tested over synthetic and real images, demonstrating the segmentation capabilities of the MGCAs obtained using the ECAS-II algorithm.

A series of application conclusions can be derived from different points of view. First, and regarding general issues, ECAS-II segmenters can operate over multi-dimensional images with different numbers of spectral bands. In this work, the MGCAs used have been evolved using RGB images. After that, they have been applied to synthetic images consisting of 64 bands, as well to hyperspectral images with different numbers of bands (115, 200 and 224 bands). In all cases, the performance results verify the applicability of the method to multi-dimensional images.

It is also important to note that the segmentation MGCAs obtained this way avoid projecting information onto lower dimensionalities during the segmentation process, which could lead to information loss in some cases. They work by modifying the whole spectrum, as shown in Figure 9 and Figure 11, which illustrate that the effect is indeed a gradual spectral modification of pixels that belong to the same region, achieving this way spectral homogenization.

On the other hand, and regarding image dependent issues, the methodology followed to tune the transition rules of the MGCA makes it possible to obtain CA segmenters that can adapt to images with different spectral and spatial features. In all the test cases, the inclusion of the ECAS-II segmenters in the processing chain improves the classification accuracy results. The amount of improvement produced by the application of the ECAS-II generated MGCAs depends on the intrinsic characteristics of the images, as well as on the type of noise or artifacts present in the hyperspectral image being processed. In this line, ECAS-II MGCA segmenters will be more efficient when spatial information is key to performing a more robust classification. This can be appreciated in Figure 15, Figure 16 and Figure 17 and Table 3 (benchmark images) and in Table 4 (images captured with our own imager). From the benchmark hyperspectral images, the Indiana scene is the noisiest and lowest-resolution image considered in the experimental section and, consequently, for this image, the inclusion of spatial information is crucial to perform an accurate segmentation and classification. Concerning the images selected from the GII-HSEG image repository, images labeled as *real_pasta_sepiolite_ID3* and *real_texture_ID12* exhibit highly-textured regions and consequently the inclusion of the segmentation step provided by the ECAS-II algorithm results in a ΔOA=14.97% and ΔOA=19.07% increase, respectively.

Additionally, the non-uniformities present in a hyperspectral image can derive from artifacts and noise in the acquisition process, changing lighting conditions or spectral mixtures. These non-uniformities are also highly relevant to the performance of the segmentation and classification process. In Section 7.1, the ECAS-II method was tested using synthetic hyperspectral images created with a high level of random noise and with a high level of spectral mixtures. In both cases, the classification performance (Table 2) is higher (ΔOA≈14%) when the ECAS-II MGCA step is introduced. These initial data indicate that performance is better in the case of spectral mixtures.

Finally, the algorithm was compared to state-of-the-art classification methods by means of its application to the segmentation of real hyperspectral images. In this respect, it has been shown that the ECAS-II + SVM algorithm provides very competitive results. 

## Figures and Tables

**Table 1:** Differential Evolution (DE) parameters.

Number of parameters:	180 (30 rules × 6 parameters/rule )
NP (Population Size):	100
CR (Crossover):	0.7
*F* (Mutation):	0.8
Stopping criterion:	Max. number of generations OR
	Min. cost
Minimum cost:	1 × 10${}^{-6}$

**Table 2:** Classification accuracies (%) for the raw pixel-wise SVM and the ECAS-II + SVM algorithm applied to the synthetic images shown in Figure 8 and Figure 10.

Image	No of Training Samples	No of Test Samples	Metric	ECAS-II + SVM (%)	SVM (%)
Figure 8	30	6370	OA	**96.96**	84.25
			AA	**93.02**	77.29
			κ	**96.65**	82.74
Figure 10	309	6091	OA	**98.56**	84.40
			AA	**98.26**	80.73
			κ	**98.47**	83.24

**Table 3:** Class specific accuracies (%) for the raw SVM and the ECAS-II + SVM algorithm in the case of segmenting the Pavia, Indiana and Salinas Scenes.

	No. of Total Samples	No. of Training Samples	Class-Specific Accuracies (%)	
			ECAS-II + SVM	SVM
PAVIA SCENE (SVM parameters: C=783.99, γ=3.70)				
1: Asphalt	6631	199	**97.65**	90.27
2: Meadows	18,649	560	**99.94**	96.67
3: Gravel	2099	63	**94.74**	73.28
4: Tree	3064	92	91.55	**92.36**
5: Metal sheets	1345	41	98.54	**99.46**
6: Base soil	5029	151	**99.45**	87.27
7: Bitumen	1330	40	**95.50**	81.47
8: Bricks	3682	111	**99.02**	83.84
9: Shadows	947	29	53.92	**99.89**
INDIANA SCENE (SVM parameters: C=512 γ=0.5)				
1: Alfalfa	46	2	**88.64**	34.09
2: Corn-notill	1428	43	**86.86**	62.53
3: Corn-mintill	830	25	**82.98**	54.78
4: Corn	237	8	**69.43**	34.06
5: Grass-pasture	483	15	**89.53**	80.34
6: Grass-trees	730	22	**95.48**	87.99
7: Grass-mowed	28	1	**70.37**	62.96
8: Hay-windrowed	478	15	**99.78**	96.11
9: Oats	20	1	**100.00**	31.58
10: Soybean-notill	972	30	**76.54**	64.86
11: Soybean-mintill	2455	74	**93.74**	74.97
12: Soybean-clean	593	18	**82.96**	45.04
13: Wheat	205	7	**98.48**	81.82
14: Woods	1265	38	**99.92**	90.46
15: Buildings-Grass	386	12	**80.21**	30.48
16: Stone-Steel-Towers	93	3	77.78	**86.67**
SALINAS SCENE (SVM parameters: C=512 γ=8)				
1: Weeds 1	2009	61	**100.00**	96.66
2: Weeds 2	3726	112	**99.94**	99.78
3: Fallow	1976	60	**100.00**	99.01
4: Fallow plow	1394	42	**99.48**	99.04
5: Fallowsmooth	2678	81	**99.50**	98.84
6: Stubble	3959	119	**99.79**	99.51
7: Celery	3579	108	**99.63**	99.39
8: Grapes	11,271	339	**94.77**	89.22
9: Soil	6203	187	**99.70**	99.42
10: Corn	3278	99	**96.45**	93.08
11: Lettuce 4wk	1068	33	**99.32**	97.00
12: Lettuce 5wk	1927	58	**100.00**	99.73
13: Lettuce 6wk	916	28	97.18	**97.75**
14: Lettuce 7wk	1070	33	89.30	**93.06**
15: Vinyard untrained	7268	219	**85.69**	69.27
16: Vinyard trellis	1807	55	**99.83**	98.29

**Table 4:** Classification accuracies (%) for the raw SVM and the ECAS-II + SVM algorithm applied to real hyperspectral images from the GII-HSEG repository.

	C	γ	No. of Training Samples	No. of Test Samples	κ (%)	OA (%)	AA (%)
real_fruit_ID1:							
SVM SVM + ECAS-II	512	0.0312	103	9884	82.76 **87.47**	87.41 **90.85**	86.18 **89.31**
real_pasta_sepiolite_ID3:							
SVM SVM + ECAS-II	512	0.5	408	19,886	71.60 **91.73**	78.86 **93.83**	81.24 **93.92**
real_texture_ID7:							
SVM SVM + ECAS-II	512	0.5	803	39,197	88.44 **91.24**	91.06 **93.18**	81.37 **90.78**
real_texture_ID9:							
SVM SVM + ECAS-II	512	0.0312	804	39,196	82.31 **92.38**	86.30 **94.10**	85.02 **93.95**
real_texture_ID12:							
SVM SVM + ECAS-II	32	2	51	908	46.07 **78.40**	68.03 **87.10**	43.92 **68.53**
